# Serum amino acid profile in patients with Parkinson’s disease

**DOI:** 10.1371/journal.pone.0191670

**Published:** 2018-01-29

**Authors:** Monika Figura, Katarzyna Kuśmierska, Ewelina Bucior, Stanisław Szlufik, Dariusz Koziorowski, Zygmunt Jamrozik, Piotr Janik

**Affiliations:** 1 Department of Neurology, Faculty of Heath Sciences, Medical University of Warsaw, Warsaw, Poland; 2 Department of Screening and Metabolic Diagnostics, Institute of Mother and Child, Warsaw, Poland; 3 1st Department of Neurology, Institute of Psychiatry and Neurology, Warsaw, Poland; 4 Department of Neurology, Medical University of Warsaw, Warsaw, Poland; University of Nebraska Medical Center, UNITED STATES

## Abstract

Amino acids play numerous roles in the central nervous system, serving as neurotransmitters, neuromodulators and regulators of energy metabolism. The free amino acid profile in serum of Parkinson’s disease (PD) patients may be influenced by neurodegeneration, mitochondrial dysfunction, malabsorption in the gastroenteric tract and received treatment. The aim of our study was the evaluation of the profile of amino acid concentrations against disease progression. We assessed the amino acid profile in the serum of 73 patients divided into groups with early PD, late PD with dyskinesia and late PD without dyskinesia. Serum amino acid analysis was performed by high-pressure liquid chromatography with fluorescence detection. We observed some significant differences amongst the groups with respect to concentrations of alanine, arginine, phenylalanine and threonine, although no significant differences were observed between patients with advanced PD with and without dyskinesia. We conclude that this specific amino acid profile could serve as biochemical marker of PD progression.

## Introduction

Parkinson’s disease is a progressive neurodegenerative disorder of unknown origin. Mitochondrial dysfunction, oxidative stress, environmental and genetic factors contribute to its development. Progressive loss of nigrostriatal dopaminergic neurons as well as noradrenergic, cholinergic and serotoninergic neurons leads to development of PD symptoms. PD patients develop problems with gastric tract motility. Increased muscle tone, dyskinesia and tremor combined with impaired digestion may lead to malnutrition [1–5]. Early amino acid-related research in PD was mainly performed with high-pressure liquid chromatography with fluorescence detection. More recently, mass spectrometry techniques have been used to analyze a larger number of amino acid compounds as well as their derivatives and dipeptides, allowing for a better understanding of this disorder [6, 7].

Changes in serum or plasma concentrations of amino acids such as glutamate, homocysteine and large neutral amino acids (LNNA-tyrosine, phenylalanine, tryptophan or branched-chain amino acids) have been reported. Homocysteine in PD may be elevated due to levodopa treatment [8]. This may be associated with PD dementia, endothelial dysfunction, increased risk of atherosclerosis and development of neuropathy in PD patients treated with levodopa [9–12]. An increase of plasma homocysteine in levodopa-treated patients is caused by the methylation of L-DOPA to 3-*orto*-methyldopa (3-OMD) in a reaction catalyzed by catechol-O-methyl transferase (COMT). This reaction requires S-adenosylmethionine (SAM) as a methyl donor group that is converted to S-adenosylhomocysteine (SAH) which is further converted to homocysteine, resulting in hyperhomocysteinemia [13]. According to some studies, other antiparkinsonian medications–COMT inhibitors–may reduce homocysteine concentration and can be applied in the clinical management of this complication [14, 15].

Glutamate and glutamine concentrations were previously assessed in PD patients. The results of studies on the concentrations of glutamate in PD were equivocal, reporting either higher glutamate concentration in serum of PD patients vs controls [16, 17] or no difference between PD patients and controls [18]. In CSF, glutamate concentration was described to be either lower in PD than in controls [18] or similar [17, 19]. Glutamate is one of three amino acids involved in synthesis of glutathione, along with cysteine and glycine. Glutathione is a tripeptide which exists in both reduced and oxidized forms. The ratio of these two forms determine the cellular redox status, which can be used as a marker of antioxidative capacity. High glutathione concentrations protect mitochondria from damage. Low glutathione concentrations may inhibit mitochondrial complex I that causes mitochondrial dysfunction and contributes to the pathogenesis of PD [20]. While some studies on the plasma levels of glutathione in PD patients vs healthy controls revealed no significant differences [21], other studies found total plasma glutathione to be lower in patients with severe PD than in controls [22]. CSF levels of glutathione are also described to be lower in PD, and the authors hypothesize that this may reflect a diminished capacity for protection against oxidative stress [23, 24]. An immediate decrease in cysteine and dipeptide cysteinyl-glycine concentrations was observed after levodopa intake, which was thought to be an indication of increased glutathione synthesis due to oxidative stress [20, 25, 26]. Post-mortem studies on glutathione levels in PD brain report reduced glutathione levels in substantia nigra [27, 28].

Levodopa is known to compete with LNAAs in crossing the blood-brain barrier by the L-type amino acid carrier [29]. It was even proven that very high concentrations of these amino acids may block up to 90–100% of levodopa from crossing the blood-brain barrier [30]. Therefore, changes in concentrations of these amino acids could possibly influence the bioavailability of levodopa. However, a study by Nutt et al. reports relatively small variation in daily plasma LNAA levels in comparison with large variations in plasma levodopa. The authors conclude that fluctuations in LNAAs are not an important contributor to the fluctuating response to levodopa [31]. A recent study by Hirayama et al. revealed high phenylalanine levels in *de novo* PD patients, but not in treated ones [32]. In contrast, tyrosine was described to be slightly lower in treated patients, but not in *de novo* patients. This difference was not significant.

The purpose of our study was to evaluate how different stages of PD and received dopaminergic treatment may affect the free amino acid profile in the serum of PD patients.

## Materials and methods

### Patients

Our study included 73 PD patients in the early stages of the disease (early PD group, ePD) or advanced stages of the disease (advanced PD group, aPD). The ePD consisted of 22 patients with unilateral or mild bilateral involvement (stage 1–2 on the Modified Hoehn and Yahr rating scale), lack of motor complications from levodopa treatment and a disease duration of less than 3 years. There were four *de novo* patients in the ePD group who were not yet receiving any PD treatment. The aPD with levodopa induced dyskinesia (aPD LID+) group consisted of 28 patients with moderate or severe bilateral involvement, motor complications and a disease duration of more than 3 years (3–5 on the Modified Hoehn and Yahr rating scale in off period). The aPD without dyskinesia (aPD LID-) consisted of 23 patients with similar clinical features but no LID. PD diagnosis was made based on UK Brain Bank Criteria. General characteristics of the group are described in Table 1.

**Table 1:** General characteristic of the study groups.

Feature	ePD (n = 22) *mean±SD*	aPD LID+ (n = 28) *mean±SD*	aPD LID- (n = 23) *mean±SD*
**Age (years)**	62.1±10.9 (range:38–84)	59.1±12.1 (range:35–80)	66±12.1 (range:29–83)
**Sex (males/females)**	11/11	11/17	13/10
**Disease duration (years)**	1.9±1.3	9.1±4.4	8.9±4.5
**LED/day**	329.8±230.6 (range: 0–700)	1090.6±434.1 (range: 160–2040)	1032.6 ±505.9 (range: 405–2240)
**UPDRS I-IV score** *	21.4±13.8	34.4±14.3	33.8±13.7

LED- levodopa equivalent dose taken daily. UPDRS- Unified Parkinson’s Disease Rating Scale. ePD- early Parkinson’s disease stadium; aPD-advanced Parkinson’s disease stadium; LID- levodopa-induced dyskinesia.

* Part III of UPDRS was assessed in “on” period.

Exclusion criteria included treatment with selegiline, rasagiline, cholinolytics, amantadine, COMT-inhibitors and neuroleptics within the past 3 months or implantation of deep brain stimulation electrodes. Only patients receiving dopaminergic treatment for PD (dopamine receptor agonists and levodopa) were included, to reduce influence of antiparkinsonian medications on amino acid concentrations. Patients with such concomitant disorders as dementia, alcohol abuse, liver damage, kidney failure, heart failure, autoimmunological disorders or other severe illnesses, as well as patients with unusual dietary habits (e.g. eliminating certain food groups from the diet) were also excluded.

The most frequently used drugs for comorbidities were ACE inhibitors and beta blockers (14% of patients included in the study, 10/73), statins (9.6%, 7/73), oral hypoglycemic agents (metformin, gliclazide– 8.2%, 6/73) and amlodipine (6.8%, 5/73). Levothyroxine, sartans and acetylsalicic acid were taken by 5.5% (4/73) of patients, and diuretics (furosemide, torasemide, hydrochlorothiazide, indapamide) and propafenone by 4.1% (3/73) of patients.

Levodopa dose equivalent (LED) was calculated by summing up the total standard release levodopa dose (milligrams) and extended-release form dose multiplied by 0.75. Dopamine agonist LED was calculated for ropinirole by multiplying drug dose in milligrams by 20, for pramipexole by 100, for piribedil by 1.

The study has been approved by the Ethics Committee of the Medical University of Warsaw (KB/81/2013), and has therefore been performed in accordance with the ethical standards laid down in the 1964 Declaration of Helsinki and its later amendments. All participants signed informed consent form prior to their inclusion in the study. Participants were recruited between November 2013 and July 2016. We approached 90 patients from the movement disorders outpatient clinics of two Medical University hospitals and 73 agreed to participate in the study.

### Sample collection

We evaluated serum basic amino acid profiles in our patients, including homocysteine, aspartic acid, glutamic acid, asparagine, serine, glutamine, glycine, threonine, citrulline, arginine, alanine, taurine, valine, methionine, tryptophan, phenylalanine, isoleucine, ornithine, leucine, lysine, proline, and tyrosine. 10 ml blood samples were obtained after 12 hours fasting and no treatment period. Only still water was allowed. The blood was taken before the morning dose of antiparkinsonian medications. aPD patients were, therefore, in their off period during blood sampling. Two aPD patients had their blood samples taken after less than 12 hours without medications, as they had very severe off periods, making them unable to visit the clinic otherwise.

Samples were centrifugated at 1500 x g for 5 min. Serum samples were aliquoted at 500 μl into Eppendorf tubes and subjected to instant freezing at -20°C. Amino acid analysis was performed successively within 3 months from blood collection.

### Analytical technique

Serum amino acid analysis was performed by high-pressure liquid chromatography reversed-phase separation and fluorescence detection (Nexera X2, Shimadzu) using the gradient method (the mobile phase 1 with 40 mmol/l phosphate buffer (pH 7.8) and the organic mobile phase 2 with 45% acetonitrile + 45% methanol + 10% water). The method described by Schwarz et al. was used with own modification for the sample preparation [33]. Own modification included deproteinization with ultrafiltration rather than methanol. After deproteinization amino acids were derivatized with o-phthalaldehyde 3-mercaptopropionic acid (OPA). 9-fluorenylmethyl chloroformate (FMOC) was added for the secondary amino acids analysis. All amino acids were detected fluorometrically. OPA and FMOC derivatization was performed on-line prior to injection. Samples were separated on Phenomenex Gemini Column (C18, 5um, 15x4,6mm). Fluorescence conditions were: excitation = 340nm and emission = 460nm absorbance.

### Statistical analysis

Statistical analysis was performed using Statistica version 13. As some of the amino acid concentrations were not normally distributed, all differences in these parameters between the three groups were assessed using The Kruskal–Wallis test by ranks and Dunn's Multiple Comparison Test. Correlations between amino acid concentrations and clinical features (disease duration, LED) were analyzed using the Spearman correlation coefficient.

## Results

**Fig 1:**
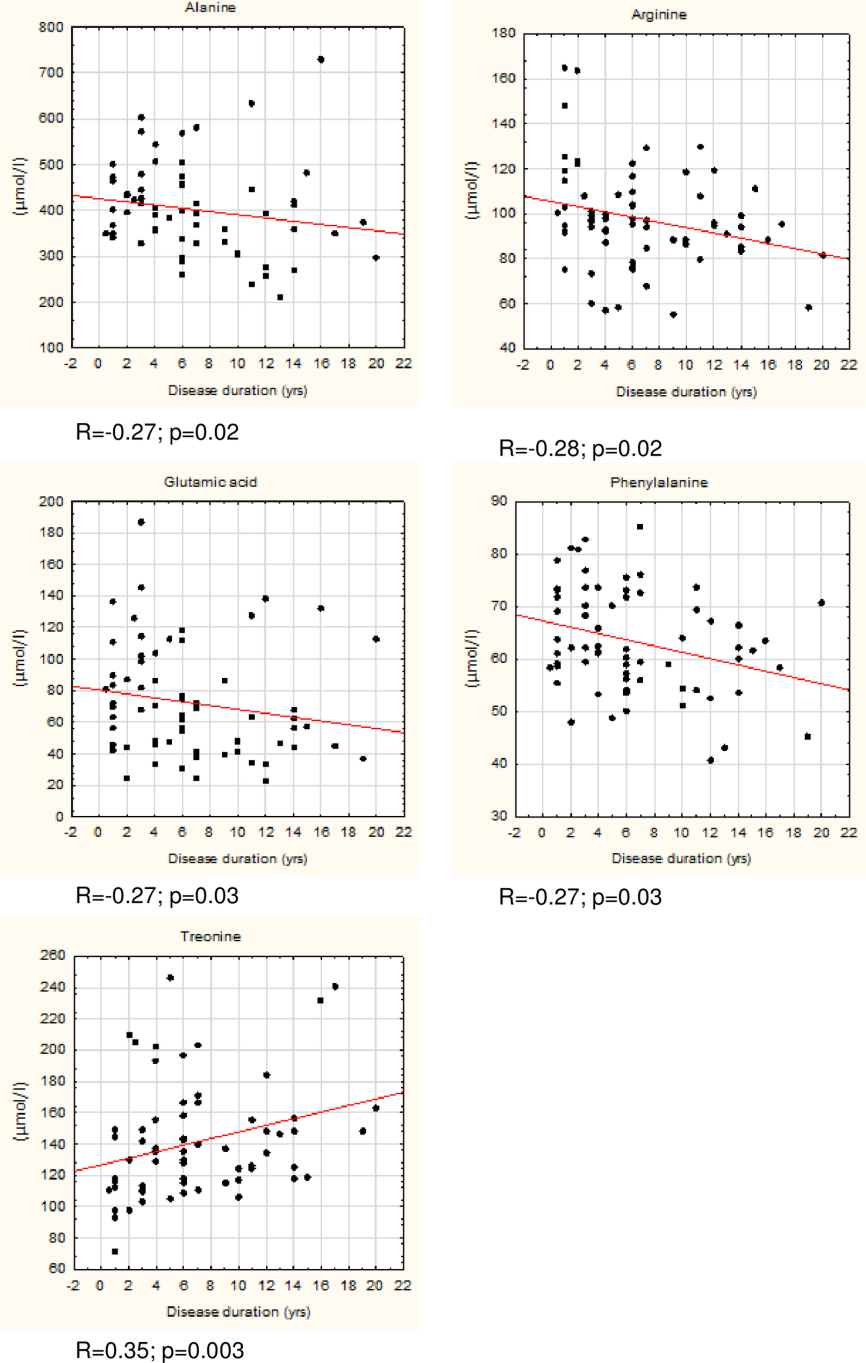
Correlations between concentrations of selected amino acids (μmol/l) and disease duration.

**Fig 2:**
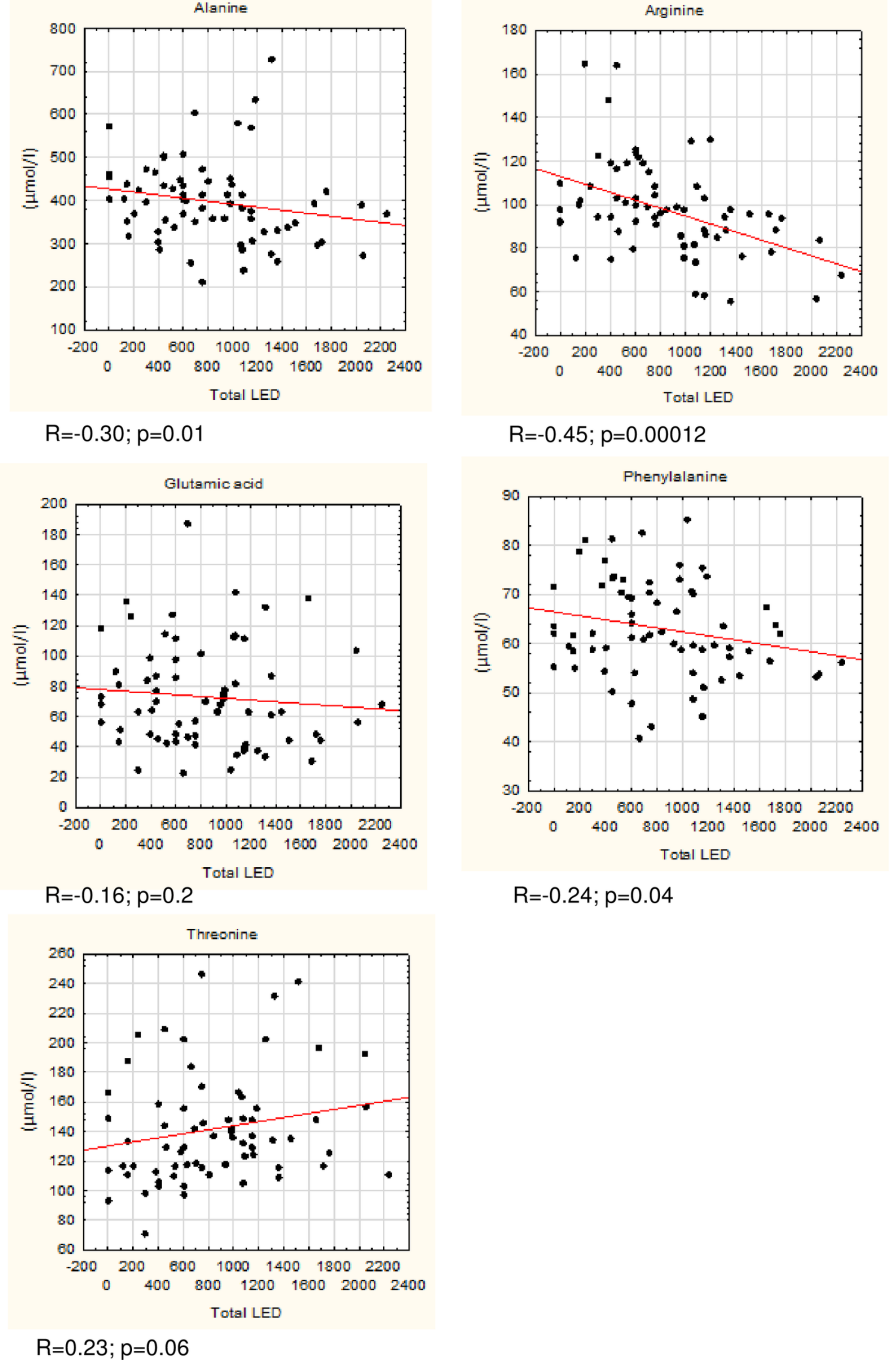
Correlations between concentrations of selected amino acids (μmol/l) and levodopa equivalent dose (LED) taken daily.

We observed significant differences in concentrations of alanine, arginine, phenylalanine, and threonine in the Kruskal-Wallis test (Table 2). No significant differences were observed regarding other amino-acids (S1 Table). Post hoc multiple comparison analysis with Dunn’s test revealed significant differences between concentrations of threonine between the ePD and aPD LID- groups (p = 0.032). Arginine concentrations differed between ePD and aPD LID+ groups (p = 0.009) and between ePD and aPD LID- groups (p = 0.047). Alanine concentration differed significantly between ePD and aPD LID+ groups (p = 0.045). Phenylalanine concentrations were significantly different between ePD and aPD LID+ groups (p = 0.005). No significant differences were observed regarding amino acid concentrations between aPD LID+ and aPD LID- groups (Table 2, S1 Table). Additionally, we calculated Spearman's rank correlation coefficient for the consolidated PD group (joint ePD, aPD LID+ and aPD LID-) to measure any correlation between amino acid concentrations and LED or disease duration. We observed that glutamate and threonine concentrations correlated with disease duration, but not with LED. The changes in concentrations of the remaining amino acids correlated with disease progression and dopaminergic treatment (Figs 1 and 2). These correlations were found to be weak but significant.

**Table 2:** Significant differences in concentrations of amino acids among three groups of PD patients compared using Kruskal-Wallis test.

Amino acid	ePD n = 22 *mean±SD* (μmol/l); median	aPD LID+ n = 28 *mean±SD* (μmol/l); median	aPD LID- n = 23 *mean±SD* (μmol/l); median	p- value
**Alanine**	430.8±70.2; 430.6	382.2±107.0; 367.1	393.5±100.4; 383.6	0.048
**Arginine**	109.4±25.4; 101.8	90.8±15.6; 92.0	92.9±19.8; 93.9	0.008
**Phenylalanine**	67.9±9.4; 69.7	59.4±8.1; 59.3	63.2±9.6; 62.1	0.008
**Threonine**	125.7±33.6; 116.6	145.0±35.1; 136.0	148.7±35.2; 148.2	0.021

ePD- early Parkinson’s disease stadium; aPD LID+ advanced Parkinson’s disease stadium with dyskinesia, aPD LID- advanced Parkinson’s disease stadium without dyskinesia.

The raw data underlying the findings of the study are presented in S2 Table.

## Discussion

Existing research on changes in concentrations of amino acids in PD with respect to its duration and treatment is sparse. A recent study by LeWitt et al. described, among other findings, a combination of 15 plasma compounds, that best predicted change in the UPDRS II+III score, including changes in serine and taurine concentrations [6]. We did not find similar results, possibly due to differences in study design. Similarly, little is known regarding changes in serum or plasma amino acid concentrations in patients developing LID. A recent study by Havelund et al. describes changes in kynurenine pathway metabolism, i.e. increased 3-HK/KYN and decreased 3HAA/ 3-HK and XA/3-HK ratios in the plasma of PD patients with LID [34]. No change in tryptophan concentration was observed, which is consistent with our results. We observed that higher serum concentrations of arginine, alanine, glutamic acid, and phenylalanine correlate with shorter disease duration and we found lower concentrations of these amino acids in patients with longer disease duration. Threonine concentrations were higher in patients with longer disease duration. Except for glutamate and threonine this change could also be related to symptomatic dopaminergic treatment (Fig 2). The possible mechanisms of amino acid serum concentration changes in PD include 1) malabsorption and changes in amino acid metabolism, 2) effects of mitochondrial dysfunction and oxidative stress, 3) reflection of progressive neurodegenerative processes in the brain, and 4) effect of dopaminergic medications and aromatic L-amino decarboxylase inhibitors.

Malabsorption of proteins in the gastroenteric tract may be caused by dysautonomia-related worsened gastroenteric motility, competition with levodopa, and/or impairment in crossing the gut-blood barrier. Higher energy expenditure in the later stages of the disease, caused by more severe parkinsonian symptoms and drug-induced dyskinesia, may also influence protein levels and lead to malnutrition. Gastric emptying in PD is delayed, which can impair the absorption of proteins and amino acid intake from food in the small intestine [4]. It was also recently described that passage through the small intestine, where absorption of amino acids takes place, is slower in PD patients than in controls [3]. However, data on actual absorption of amino acids in PD is equivocal. It was described that absorption of phenylalanine and tyrosine is compromised in PD subjects compared with age-matched controls [35]. On the other hand, a study performed by Granerus et al. provided contradictory results, revealing no difference in absorption and elimination of these two amino acids between PD and control patients [36]. It would also be difficult to explain all changes in amino acid profile by referring to an increase in malabsorption with PD progression, as we observed no change in the levels of many amino acids in aPD compared to ePD (S1 Table). The finding that only some amino acid concentrations change with respect to the stage of the disease suggests that dopaminergic treatment and/or disease progression may selectively affect their absorption and elimination, depending on their biochemical properties and role in protein synthesis.

We also found that the concentration of threonine was higher among aPD patients and that this was not related to dopaminergic treatment (p = 0.06) but dependent on disease duration (Figs 1 and 2). Threonine has an established role in the synthesis of mucins, proteins that form a gel-like protective barrier on the intestine walls. It was described on the basis of pig models that up to 60% of threonine is retained in the gastroenteric tract to form mucins [37]. We can only speculate that a gradual increase in serum threonine may be related to a disruption of the mucin layer. Another explanation involves the role of threonine as an indicator of undernutrition. Threonine is an essential exogenous amino acid that helps to maintain a proper protein balance in the body. Threonine concentration decreases with limited supply of protein and increases during prolonged fasting [38, 39]. Undernutrition is especially linked to more advanced PD, as rated on the Hoehn and Yahr and UPDRS scales [2]. A study by Jaafar et al. found that almost a quarter of their PD patients’ cohort was at medium or high risk of malnutrition [1].

We found lower serum concentrations of arginine in late PD LID+ and LID- patients compared to the early PD group. We can speculate that a shift in arginine concentrations may also be linked to oxidative stress. The y+ system transports arginine into the CNS, enabling nitric oxide (NO) synthesis [40]. Thus, a reduction of seral arginine concentrations with PD duration may reflect higher NO synthesis and greater nitrative stress in our cohort. This is in line with a study by Kirbas et al., which showed that NO levels are higher in PD patients compared to healthy controls [41]. It is known that nitrative and oxidative stress contribute to neurodegeneration in PD [42].

Changes in glutamic acid concentrations seem to be of interest, as this amino acid plays a role as a neurotransmitter in the brain and is involved in the pathogenesis of PD and development of LID. We observed a weak, but significant negative correlation between glutamate concentration and duration of the disease. Some authors suggest that increased serum concentrations of glutamate may reflect increased cerebral glutamatergic activity [43, 44]. Glutamate is also involved in synthesis of glutathione as well as brain energy metabolism. Decreased extracellular glutamate concentrations were observed by Lei et al. in ex vivo models of parkinsonism induced by paraquat, MPP^+^, and rotenone [45]. The authors hypothesize that glutathione synthesis is directly dependent on glucose metabolism, which correlates with a decrease in the content of the glutathione precursor glutamate. These findings suggest that glutamate levels may be reduced with an increase in oxidative stress as the disease progresses. Other studies on this subject presented contradictory findings of increased concentrations of glutamate in lesioned striata [46, 47]. A study by Iwasaki reported increased glutamate concentration in the plasma of PD patients versus healthy controls, however other studies did not support this finding [17, 18]. It is not clear if changes in striatal levels of glutamate are reflected in plasma or serum.

A study by Wuolikainen et al. emphasizes the role of metabolites of branched- chained amino acids, especially leucine, in maintaining glutamate homeostasis in the brain. It also mentions increased levels of alanine in the plasma and CSF of PD patients [48]. A consistent finding of an increase in concentration of alanine in the plasma and CSF of PD patients, as well as serine and methionine was described in a study performed by Trupp et al., and these authors suggest that changes in concentrations of amino acids and fatty acids reflect altered energy metabolism in PD [7]. In our study we observed alanine levels in serum to be lower in the aPD LID+ group than in the ePD group.

Data on amino acid pattern in the urine of PD patients is scarce. Metabolomic studies conducted on urine report, among others, alterations in tryptophan, glycine and phenylalanine metabolism [49, 50]. Levodopa metabolites are elevated in the urine of treated PD patients [51].

We conclude that a specific pattern of serum free amino acids including differences in concentrations of phenylalanine, arginine, alanine and threonine differs between early and advanced PD. Changes in glutamate and arginine concentrations may reflect neurodegenerative process of PD.

### Limitations

Considering the many factors which may influence amino acid concentrations, such as diet, concomitant disorders, and possible changes in metabolic pathways related to the disease, our findings need further validation, especially regarding potential alterations in energy metabolism in PD patients. It seems crucial for future studies to include different tissue and fluid samples (urine, plasma, CSF) from one patient, which will allow for a more comprehensive analysis.

## Supporting information

S1 Table Non-significant differences in concentrations of amino acids among three groups of PD patients compared in Kruskal-Wallis test. ePD- early Parkinson’s disease stadium; aPD LID+ advanced Parkinson’s disease stadium with dyskinesia, aPD LID- advanced Parkinson’s disease stadium without dyskinesia.
(DOCX)

S2 Table Raw data underlying the findings of the study. ePD- early Parkinson’s disease stadium; aPD LID+ advanced Parkinson’s disease stadium with dyskinesia, aPD LID- advanced Parkinson’s disease stadium without dyskinesia.
(XLSX)

## References

[1] Jaafar AF, Gray WK, Porter B, Turnbull EJ, Walker RW. A cross-sectional study of the nutritional status of community-dwelling people with idiopathic Parkinson's disease. BMC neurology. 2010;10(1):1. 21192784 10.1186/1471-2377-10-124 PMC3022770

[2] Sheard JM, Ash S, Mellick GD, Silburn PA, Kerr GK. Malnutrition in a sample of community-dwelling people with Parkinson’s disease. PLoS One. 2013;8(1):e53290 doi: 10.1371/journal.pone.0053290 23326408 10.1371/journal.pone.0053290 PMC3541272

[3] Dutkiewicz J, Szlufik S, Nieciecki M, Charzyńska I, Królicki L, Smektała P, et al Small intestine dysfunction in Parkinson’s disease. Journal of Neural Transmission. 2015;122(12):1659–61. doi: 10.1007/s00702-015-1442-0 26306670 10.1007/s00702-015-1442-0 PMC4644198

[4] Edwards LL, Quigley EM, Pfeiffer RF. Gastrointestinal dysfunction in Parkinson's disease: frequency and pathophysiology. Neurology. 1992;42(4):726–32. 1565224 10.1212/wnl.42.4.726

[5] Bestetti A, Capozza A, Lacerenza M, Manfredi L, Mancini F. Delayed Gastric Emptying in Advanced Parkinson Disease: Correlation With Therapeutic Doses. Clinical nuclear medicine. 2017;42(2):83–7. doi: 10.1097/RLU.0000000000001470 27941374 10.1097/RLU.0000000000001470

[6] LeWitt PA, Li J, Lu M, Guo L, Auinger P. Metabolomic biomarkers as strong correlates of Parkinson disease progression. Neurology. 2017;88(9):862–9. doi: 10.1212/WNL.0000000000003663 28179471 10.1212/WNL.0000000000003663 PMC5331866

[7] Trupp M, Jonsson P, Öhrfelt A, Zetterberg H, Obudulu O, Malm L, et al Metabolite and peptide levels in plasma and CSF differentiating healthy controls from patients with newly diagnosed Parkinson's disease. Journal of Parkinson's disease. 2014;4(3):549–60. doi: 10.3233/JPD-140389 24927756 10.3233/JPD-140389

[8] Kuhn W, Roebroek R, Blom H, Van Oppenraaij D, Przuntek H, Kretschmer A, et al Elevated Plasma Levels of Homocysteine inParkinson’s Disease. European neurology. 1998;40(4):225–7. 9813406 10.1159/000007984

[9] Müller T, Renger K, Kuhn W. Levodopa-associated increase of homocysteine levels and sural axonal neurodegeneration. Archives of Neurology. 2004;61(5):657–60. doi: 10.1001/archneur.61.5.657 15148140 10.1001/archneur.61.5.657

[10] Yoon JH, Lee JS, Yong SW, Hong JM, Lee PH. Endothelial dysfunction and hyperhomocysteinemia in Parkinson's disease: Flow‐mediated dilation study. Movement Disorders. 2014;29(12):1551–5. doi: 10.1002/mds.26005 25154960 10.1002/mds.26005

[11] Szadejko K, Dziewiatowski K, Szabat K, Robowski P, Schinwelski M, Sitek E, et al Polyneuropathy in levodopa-treated Parkinson's patients. Journal of the Neurological Sciences. 2016;371:36–41. doi: 10.1016/j.jns.2016.09.061 27871444 10.1016/j.jns.2016.09.061

[12] Zoccolella S, Abruzzese G, Antonini A, Bonuccelli U, Canesi M, Cristina S, et al Hyperhomocysteinemia in levodopa‐treated patients with Parkinson's disease dementia. Movement Disorders. 2009;24(7):1028–33. doi: 10.1002/mds.22511 19353704 10.1002/mds.22511

[13] Sharma M, Tiwari M, Tiwari RK. Hyperhomocysteinemia: impact on neurodegenerative diseases. Basic & clinical pharmacology & toxicology. 2015;117(5):287–96. 26036286 10.1111/bcpt.12424

[14] Müller T, Kuhn W. Tolcapone decreases plasma levels of S-adenosyl-L-homocysteine and homocysteine in treated Parkinson’s disease patients. European journal of clinical pharmacology. 2006;62(6):447–50. doi: 10.1007/s00228-006-0132-0 16758261 10.1007/s00228-006-0132-0

[15] Valkovič P, Benetin J, Blažíček P, Gmitterová K, Kukumberg P. Reduced plasma homocysteine levels in levodopa/entacapone treated Parkinson patients. Parkinsonism & related disorders. 2005;11(4):253–6. 15878587 10.1016/j.parkreldis.2005.01.007

[16] Iwasaki Y, Ikeda K, Shiojima T, Kinoshita M. Increased plasma concentrations of aspartate, glutamate and glycine in Parkinson's disease. Neuroscience letters. 1992;145(2):175–7. 1361223 10.1016/0304-3940(92)90015-y

[17] Jiménez-Jiménez FJ, Molina J, Vargas C, Gómez P, Navarro J, Benito-Leon J, et al Neurotransmitter amino acids in cerebrospinal fluid of patients with Parkinson's disease. Journal of the neurological sciences. 1996;141(1):39–44. 8880690 10.1016/0022-510x(96)00115-3

[18] Mally J, Szalai G, Stone T. Changes in the concentration of amino acids in serum and cerebrospinal fluid of patients with Parkinson's disease. Journal of the neurological sciences. 1997;151(2):159–62. 9349670 10.1016/s0022-510x(97)00119-6

[19] Kuiper M, Teerlink T, Visser J, Bergmans P, Scheltens P, Wolters EC. L-glutamate, L-arginine and L-citrulline levels in cerebrospinal fluid of Parkinson's disease, multiple system atrophy, and Alzheimer's disease patients. Journal of neural transmission. 2000;107(2):183–9. doi: 10.1007/s007020050016 10847559 10.1007/s007020050016

[20] Müller T, Trommer I, Muhlack S, Mueller BK. Levodopa increases oxidative stress and repulsive guidance molecule A levels: a pilot study in patients with Parkinson’s disease. Journal of Neural Transmission. 2016;123(4):401–6. doi: 10.1007/s00702-016-1519-4 26880022 10.1007/s00702-016-1519-4

[21] Watfa G, Dragonas C, Brosche T, Dittrich R, Sieber C, Alecu C, et al Study of telomere length and different markers of oxidative stress in patients with Parkinson’s disease. The journal of nutrition, health & aging. 2011;15(4):277–81. 10.1007/s12603-010-0275-7 PMC12879577 21437559

[22] Cristalli DO, Arnal N, Marra FA, de Alaniz MJ, Marra CA. Peripheral markers in neurodegenerative patients and their first-degree relatives. Journal of the Neurological Sciences. 2012;314(1):48–56. 22113180 10.1016/j.jns.2011.11.001

[23] Maetzler W, Schmid SP, Wurster I, Liepelt I, Gaenslen A, Gasser T, et al Reduced but not oxidized cerebrospinal fluid glutathione levels are lowered in Lewy body diseases. Movement Disorders. 2011;26(1):176–81. doi: 10.1002/mds.23358 20842692 10.1002/mds.23358

[24] LeWitt PA, Li J, Lu M, Beach TG, Adler CH, Guo L. 3‐hydroxykynurenine and other Parkinson's disease biomarkers discovered by metabolomic analysis. Movement Disorders. 2013;28(12):1653–60. doi: 10.1002/mds.25555 23873789 10.1002/mds.25555

[25] Müller T, Muhlack S. Cysteinyl‐glycine reduction as marker for levodopa‐induced oxidative stress in Parkinson's disease patients. Movement Disorders. 2011;26(3):543–6. doi: 10.1002/mds.23384 21462263 10.1002/mds.23384

[26] Müller T, Muhlack S. Cysteine decrease following acute Levodopa intake in patients with Parkinson's disease. Neuroscience letters. 2012;521(1):37–9. doi: 10.1016/j.neulet.2012.05.054 22641055 10.1016/j.neulet.2012.05.054

[27] Kish SJ, Morito C, Hornykiewicz O. Glutathione peroxidase activity in Parkinson's disease brain. Neuroscience letters. 1985;58(3):343–6. 4047494 10.1016/0304-3940(85)90078-3

[28] Sian J, Dexter DT, Lees AJ, Daniel S, Agid Y, Javoy‐Agid F, et al Alterations in glutathione levels in Parkinson's disease and other neurodegenerative disorders affecting basal ganglia. Annals of neurology. 1994;36(3):348–55. doi: 10.1002/ana.410360305 8080242 10.1002/ana.410360305

[29] Nutt JG, Woodward WR, Hammerstad JP, Carter JH, Anderson JL. The on–off phenomenon in Parkinson's disease: relation to levodopa absorption and transport. New England Journal of Medicine. 1984;310(8):483–8. doi: 10.1056/NEJM198402233100802 6694694 10.1056/NEJM198402233100802

[30] Kageyama T, Nakamura M, Matsuo A, Yamasaki Y, Takakura Y, Hashida M, et al The 4F2hc/LAT1 complex transports L-DOPA across the blood–brain barrier. Brain research. 2000;879(1):115–21. 11011012 10.1016/s0006-8993(00)02758-x

[31] Nutt J, Woodward W, Carter J, Trotman T. Influence of fluctuations of plasma large neutral amino acids with normal diets on the clinical response to levodopa. Journal of Neurology, Neurosurgery & Psychiatry. 1989;52(4):481–7. 10.1136/jnnp.52.4.481 PMC1032296 2738591

[32] Hirayama M, Tsunoda M, Yamamoto M, Tsuda T, Ohno K. Serum tyrosine-to-phenylalanine ratio is low in Parkinson’s disease. Journal of Parkinson's disease. 2016;(Preprint):1–9. 10.3233/JPD-150736 27061063

[33] Schwarz EL, Roberts WL, Pasquali M. Analysis of plasma amino acids by HPLC with photodiode array and fluorescence detection. Clinica Chimica Acta. 2005;354(1):83–90. 10.1016/j.cccn.2004.11.016 15748603

[34] Havelund JF, Andersen AD, Binzer M, Blaabjerg M, Heegaard NH, Stenager E, et al Changes in kynurenine pathway metabolism in Parkinson patients with L‐dopa‐induced dyskinesia. Journal of Neurochemistry. 2017;142(5):756–66. doi: 10.1111/jnc.14104 28628213 10.1111/jnc.14104

[35] Braham J, Sarova-Pinhas I, Crispin M, Golan R, Levin N, Szeinberg A. Oral phenylalanine and tyrosine tolerance tests in Parkinsonian patients. British medical journal. 1969;2(5656):552 5769891 10.1136/bmj.2.5656.552 PMC1983481

[36] Granerus A-K, Jagenburg R, Rödjer S, Svanborg A. Phenylalanine absorption and metabolism in parkinsonian patients. Br Med J. 1971;4(5782):262–4. 5123908 10.1136/bmj.4.5782.262 PMC1799570

[37] Stoll B, Burrin DG, Henry J, Yu H, Jahoor F, Reeds PJ. Substrate oxidation by the portal drained viscera of fed piglets. American Journal of Physiology-Endocrinology And Metabolism. 1999;277(1):E168–E75. 10.1152/ajpendo.1999.277.1.E168 10409141

[38] Blom W, Huijmans J. Differential diagnosis of (inherited) amino acid metabolism or transport disorders. Amino acids. 1992;2(1–2):25–67. doi: 10.1007/BF00806075 24194272 10.1007/BF00806075

[39] Felig P, Owen OE, Wahren J, Cahill GF Jr. Amino acid metabolism during prolonged starvation. Journal of Clinical Investigation. 1969;48(3):584 doi: 10.1172/JCI106017 5773094 10.1172/JCI106017 PMC535724

[40] O’Kane RL, Vina JR, Simpson I, Zaragozá R, Mokashi A, Hawkins RA. Cationic amino acid transport across the blood-brain barrier is mediated exclusively by system y+. American Journal of Physiology-Endocrinology and Metabolism. 2006;291(2):E412–E9. doi: 10.1152/ajpendo.00007.2006 16569760 10.1152/ajpendo.00007.2006

[41] Kirbas S, Kirbas A, Tufekci A, Cumhur Cure M, Cakmak S, Yazici T, et al Serum levels of homocysteine, asymmetric dimethylarginine and nitric oxide in patients with Parkinson’s disease. Acta Clinica Belgica. 2016;71(2):71–5. doi: 10.1080/17843286.2016.1138592 27075796 10.1080/17843286.2016.1138592

[42] Jenner P, Dexter D, Sian J, Schapira A, Marsden C. Oxidative stress as a cause of nigral cell death in Parkinson's disease and incidental Lewy body disease. Annals of Neurology. 1992;32(S1):S82–S7. 1510385 10.1002/ana.410320714

[43] Shimmura C, Suda S, Tsuchiya KJ, Hashimoto K, Ohno K, Matsuzaki H, et al Alteration of plasma glutamate and glutamine levels in children with high-functioning autism. PLoS One. 2011;6(10):e25340 doi: 10.1371/journal.pone.0025340 21998651 10.1371/journal.pone.0025340 PMC3187770

[44] Shinohe A, Hashimoto K, Nakamura K, Tsujii M, Iwata Y, Tsuchiya KJ, et al Increased serum levels of glutamate in adult patients with autism. Progress in Neuro-Psychopharmacology and Biological Psychiatry. 2006;30(8):1472–7. doi: 10.1016/j.pnpbp.2006.06.013 16863675 10.1016/j.pnpbp.2006.06.013

[45] Lei S, Zavala-Flores L, Garcia-Garcia A, Nandakumar R, Huang Y, Madayiputhiya N, et al Alterations in energy/redox metabolism induced by mitochondrial and environmental toxins: a specific role for glucose-6-phosphate-dehydrogenase and the pentose phosphate pathway in paraquat toxicity. ACS chemical biology. 2014;9(9):2032 doi: 10.1021/cb400894a 24937102 10.1021/cb400894a PMC4168797

[46] Gao H-C, Zhu H, Song C-Y, Lin L, Xiang Y, Yan Z-H, et al Metabolic changes detected by ex vivo high resolution 1H NMR spectroscopy in the striatum of 6-OHDA-induced Parkinson’s rat. Molecular neurobiology. 2013;47(1):123–30. doi: 10.1007/s12035-012-8336-z 22936308 10.1007/s12035-012-8336-z

[47] Chassain C, Bielicki G, Durand E, Lolignier S, Essafi F, Traoré A, et al Metabolic changes detected by proton magnetic resonance spectroscopy in vivo and in vitro in a murin model of Parkinson’s disease, the MPTP‐intoxicated mouse. Journal of neurochemistry. 2008;105(3):874–82. doi: 10.1111/j.1471-4159.2007.05185.x 18088356 10.1111/j.1471-4159.2007.05185.x

[48] Wuolikainen A, Jonsson P, Ahnlund M, Antti H, Marklund SL, Moritz T, et al Multi-platform mass spectrometry analysis of the CSF and plasma metabolomes of rigorously matched amyotrophic lateral sclerosis, Parkinson's disease and control subjects. Molecular BioSystems. 2016;12(4):1287–98. doi: 10.1039/c5mb00711a 26883206 10.1039/c5mb00711a

[49] Luan H, Liu L-F, Meng N, Tang Z, Chua K-K, Chen L-L, et al LC–MS-based urinary metabolite signatures in idiopathic Parkinson’s disease. Journal of proteome research. 2014;14(1):467–78. doi: 10.1021/pr500807t 25271123 10.1021/pr500807t

[50] Luan H, Liu L-F, Tang Z, Zhang M, Chua K-K, Song J-X, et al Comprehensive urinary metabolomic profiling and identification of potential noninvasive marker for idiopathic Parkinson’s disease. Scientific reports. 2015;5. 10.1038/srep13888 PMC4568456 26365159

[51] Eisenhofer G, Brown S, Peitzsch M, Pelzel D, Lattke P, Glöckner S, et al Levodopa therapy in Parkinson’s disease: influence on liquid chromatographic tandem mass spectrometric-based measurements of plasma and urinary normetanephrine, metanephrine and methoxytyramine. Annals of clinical biochemistry. 2014;51(1):38–46. 23873873 10.1177/0004563213487894

