# Supplementary material for: Serum amino acid profile in patients with Parkinson’s disease
Source: PLoS One. 2018 Jan 29;13(1):e0191670. doi: 10.1371/journal.pone.0191670 (PMC5788376; doi:10.1371/journal.pone.0191670)
Supplement: S1 Table — ePD- early Parkinson’s disease stadium; aPD LID+ advanced Parkinson’s disease stadium with dyskinesia, aPD LID- advanced Parkinson’s disease stadium without dyskinesia. (DOCX) [file pone.0191670.s001.docx]

| **Amino acid** | **ePD**  **n=22**  **mean±SD**  **(µmol/l); median** | **aPD LID+**  **n=28**  **mean±SD**  **(µmol/l)**  **median** | **aPD LID-**  **n=23**  **mean±SD**  **(µmol/l)**  **median** | **p- value** |
| --- | --- | --- | --- | --- |
| **Asparagine** | 52.4±8.9; 52.2 | 51.7±8.2; 51.4 | 50.1±134; 50.3 | 0.86 |
| **Aspartic acid** | 24.5±10.8; 25.6 | 18.8±7.8;18.2 | 17.9±5,6; 16.4 | 0.12 |
| \| **Citrulline** \| \| --- \| | 37.6±8.7; 35.7 | 35.2±8.5;33.3 | 37.0±8,3; 37.4 | 0.3 |
| **Glutamic acid** | 86.8±40.0; 82.6 | 69.4±31.8; 62.0 | 64.0± 29.0; 57.1 | 0.11 |
| \| **Glutamine** \| \| --- \| | 593.5±65.0; 605.1 | 602.7±72.8; 600.6 | 602.2±93.7; 574.4 | 0.66 |
| \| **Glycine** \| \| --- \| | 296.3±77.0; 273.1 | 373.3±151.4; 322.7 | 320.7±87.8; 305.3 | 0.22 |
| \| **Homocysteine** \| \| --- \| | 13.3±3.6; 13.0 | 14.5±7.8; 12.8 | 13.8±5.0; 11.9 | 0.87 |
| \| **Isoleucine** \| \| --- \| | 66.8±13.7; 64 | 62.2±12.3; 61.8 | 65.5±15.1; 61.9 | 0.66 |
| \| **Leucine** \| \| --- \| | 132.0±21.5;130.1 | 124.1±27.1; 117.2 | 129.1±24.7; 121.7 | 0.27 |
| \| **Lysine** \| \| --- \| | 187.2±40.1; 181.7 | 174.0±36.7; 167.0 | 183.6±33.6; 183.1 | 0.38 |
| \| **Methionine** \| \| --- \| | 23.5±3.6; 23.9 | 21.3±3.2; 21.5 | 22.8±5.6; 22.3 | 0.13 |
| \| **Ornitine** \| \| --- \| | 96.8±30.3; 100.8 | 108.0±32.8; 102.1 | 114.6± 37.9; 109,.6 | 0.36 |
| \| **Proline** \| \| --- \| | 200.5±64.0; 177.0 | 188.2±62.1;186.5 | 193.9±66.6;176.2 | 0.80 |
| **Serine** | 132.3±26.2; 128.55 | 128.6±22.1; 125.1 | 125.6±17.8;122.9 | 0.67 |
| \| **Taurine** \| \| --- \| | 118.3±45.6; 125.8 | 115.3±48.7; 108.2 | 106.3±38.1; 95.1 | 0.76 |
| \| **Tryptophan** \| \| --- \| | 33.1±8.2; 32.1 | 30.6±7.8; 32.9 | 34.3±8.5; 34.9 | 0.24 |
| \| **Tyrosine** \| \| --- \| | 67.9±15.3; 15.3 | 71.5±21.7; 67.0 | 76.1±22.7; 75.1 | 0.44 |
| \| **Valine** \| \| --- \| | 225.2±43.8; 221.4 | 206.5±38.2; 203.7 | 226,5±44,5; 214.4 | 0.28 |

S1 Table. **Non-significant differences in concentrations of amino acids among three groups of PD patients compared in Kruskal-Wallis test.** ePD- early Parkinson’s disease stadium; aPD-advanced Parkinson’s disease stadium; LID- levodopa-induced dyskinesia.
